# Basilic vein aneurysm and palmar intramuscular hemangioma with peri and inter tendinous growth^☆^

**DOI:** 10.1016/j.radcr.2022.05.078

**Published:** 2022-07-02

**Authors:** Farzaneh Shobeirian, Zahra Ghomi, Mohammad Zare Mehrjardi, Nikan Zerafatjou

**Affiliations:** a Department of Radiology, Razi Hospital, Guilan University of Medical Sciences, Rasht, Iran; b Department of Radiology, Mofid Children's Hospital, School of Medicine, Shahid Beheshti University of Medical Sciences, Tehran, Iran; c Department of Radiology, Shohada-e-Tajrish Hospital, Shahid Beheshti University of Medical Sciences, Tehran, Iran; d Department of Urology, Shahid Rajaee Hospital, Isfahan University of Medical Sciences, Isfahan, Iran

**Keywords:** Veins, Aneurysm, Hemangioma, Palmar mass

## Abstract

In this report, we describe a case of concomitant basilic vein aneurysm and palmar hemangioma with peri- and inter-tendinous growth around the fourth and fifth flexor digitorum superficialis and flexor digitorum profundus tendons.

It seems reasonable for physicians and radiologists to keep in mind the possibility of venous aneurysms in the presence of soft tissue hemangiomas; as they can present as palpable mass and be mistaken for other pathologies. Familiarity with clinical and imaging findings of this entity could be helpful to prevent misdiagnosis.

## Introduction

Venous aneurysm (VA) - defined as focal saccular or fusiform dilatation of a vein- is an uncommon clinical entity; and is classified based on etiology as primary or secondary. Secondary venous aneurysms are caused by trauma, infection, venous valve insufficiency or in association with hemodialysis fistulas. Primary venous aneurysms are attributed to a congenital defect in walls of a vein. This type of venous aneurysms are very rare; especially in superficial veins, and can be a source of diagnostic challenge [1].

**Fig. 1:**
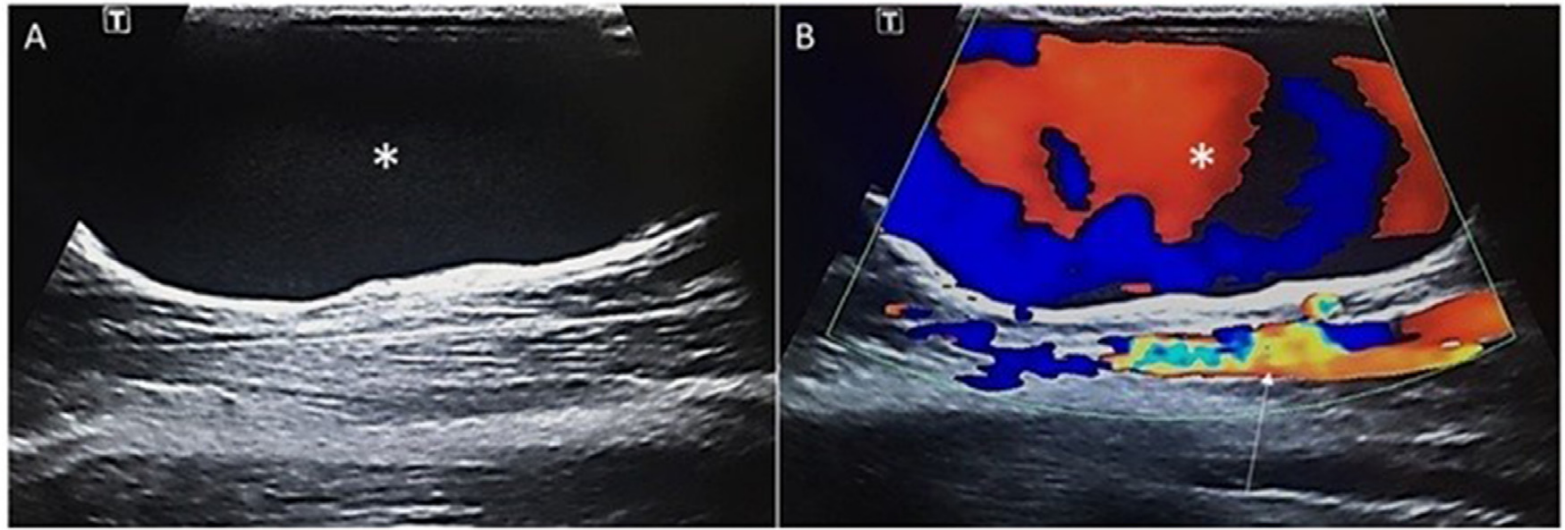
Grayscale (A) and color Doppler (B) ultrasound of the basilic vein demonstrates fusiform aneurysmal dilatation of basilic vein (asterisk) is noted with a slow turbulent flow in color Doppler study. The ulnar artery is seen deep to the Aneurysmally dilated basilic vein (arrow).

**Fig. 2:**
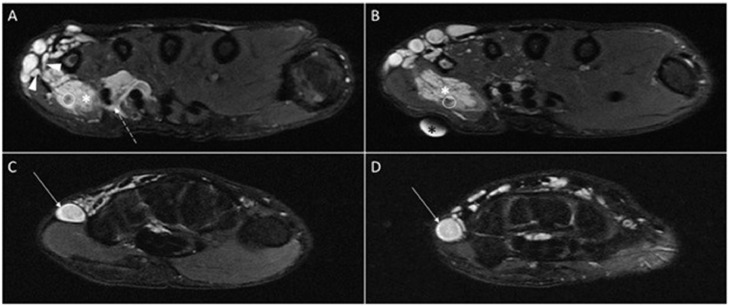
Axial fat-suppressed proton density (PD) MRI of hand and wrist. (A, B) A lobulated hyperintense mass (white asterisk) is seen in the hypothenar region at the site of lump palpation (black asterisk indicates the body marker). Peri and inter tendinous growth are noted around the fourth and fifth flexor digitorum superficialis and profundus tendons (dashed arrow). The mass contains internal areas of hypo signal intensity (circles) indicating phleboliths. Dilated tortuous draining veins are seen medially (arrowheads). (C, D) The aneurysmally dilated basilic vein is seen in the medial aspect of the forearm with a turbulent flow (arrow). Engorgement of other adjacent superficial veins is noted as well.

Basilic vein aneurysm has been reported in very few cases [1], [2], [3], [4], [5] and its co-occurrence with deep hemangiomas has been reported only in one case [6]. In this report, we present a case with co-occurrence of palmar intramuscular hemangioma and basilic vein aneurysm in distal forearm; with emphasis on preoperative diagnostic workup and imaging findings.

## Case presentation

A 35-year-old woman presented to general surgery clinic with complaint of slowly-growing mass on her distal forearm, which has grown over the previous year. She also had noticed a painless, stable-size palpable mass on the palm of her hand since over four years ago. Patient had no history of trauma or any cannulation at any time.

On physical examination, a tubular mass-like structure was visible in the medial aspect of distal forearm. There was also a palpable, soft, fluctuant and nontender mass in the hypothenar region at the site of lump palpation. The masses were not pulsatile or tender.

On grayscale and color Doppler ultrasonographic examination, an aneurysmally dilated basilic vein was noted in the medial aspect of the distal forearm; with a 22 mm diameter and slow turbulent flow (Fig. 1). There was also an echogenic mass with scattered calcified foci in the hypothenar region at the site of lump palpation. Scattered areas of minimal blood flow within the mass were seen on color Doppler study.

**Fig. 3:**
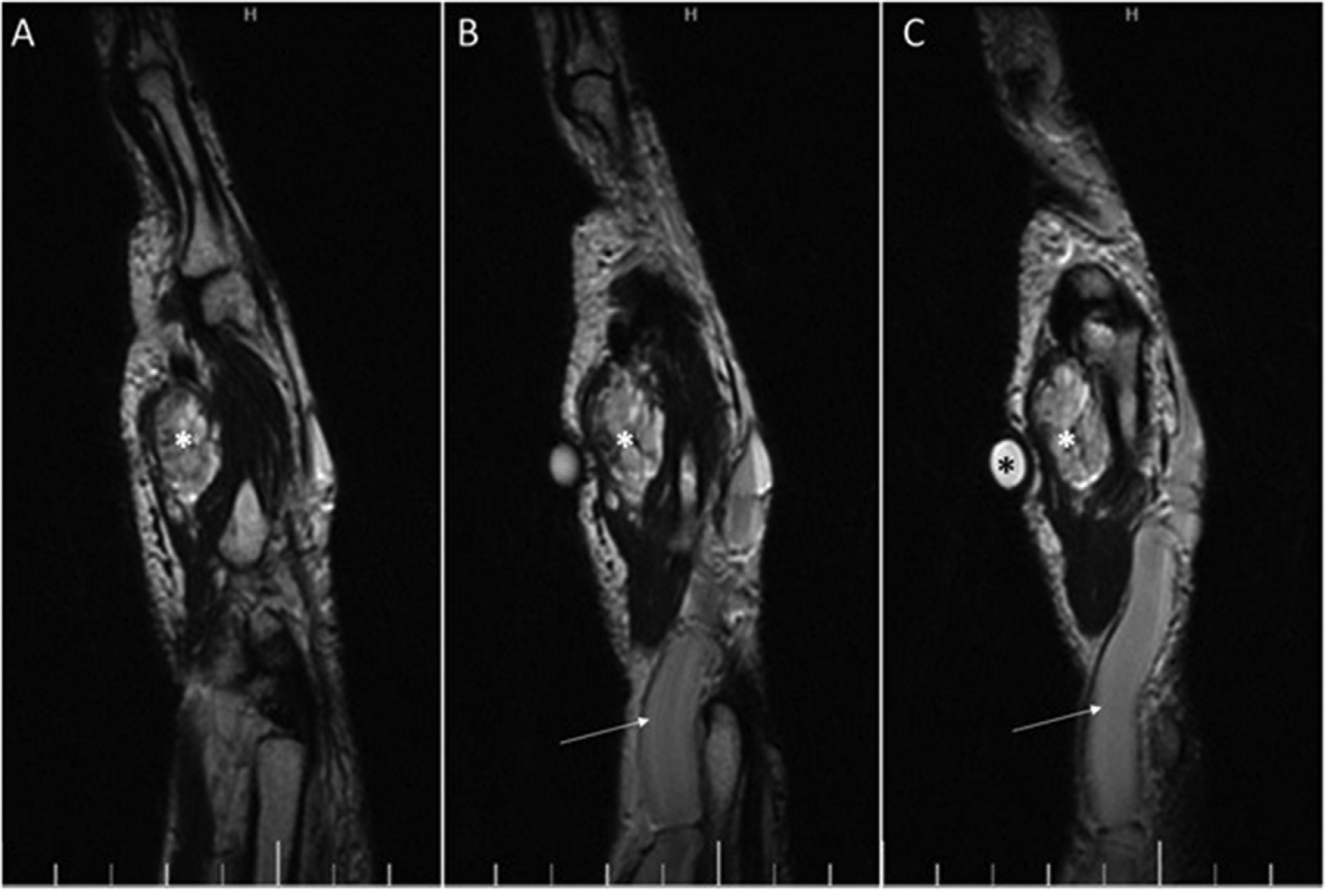
Sagittal T2 weighted MRI of wrist and hand. (A-C) Hyper signal soft tissue mass (white asterisk) is seen in the palmar aspect of the hand at the site of lump palpation (black asterisk indicates the body marker). The aneurysmal dilatation of basilic vein is noted (arrow).

**Fig. 4:**
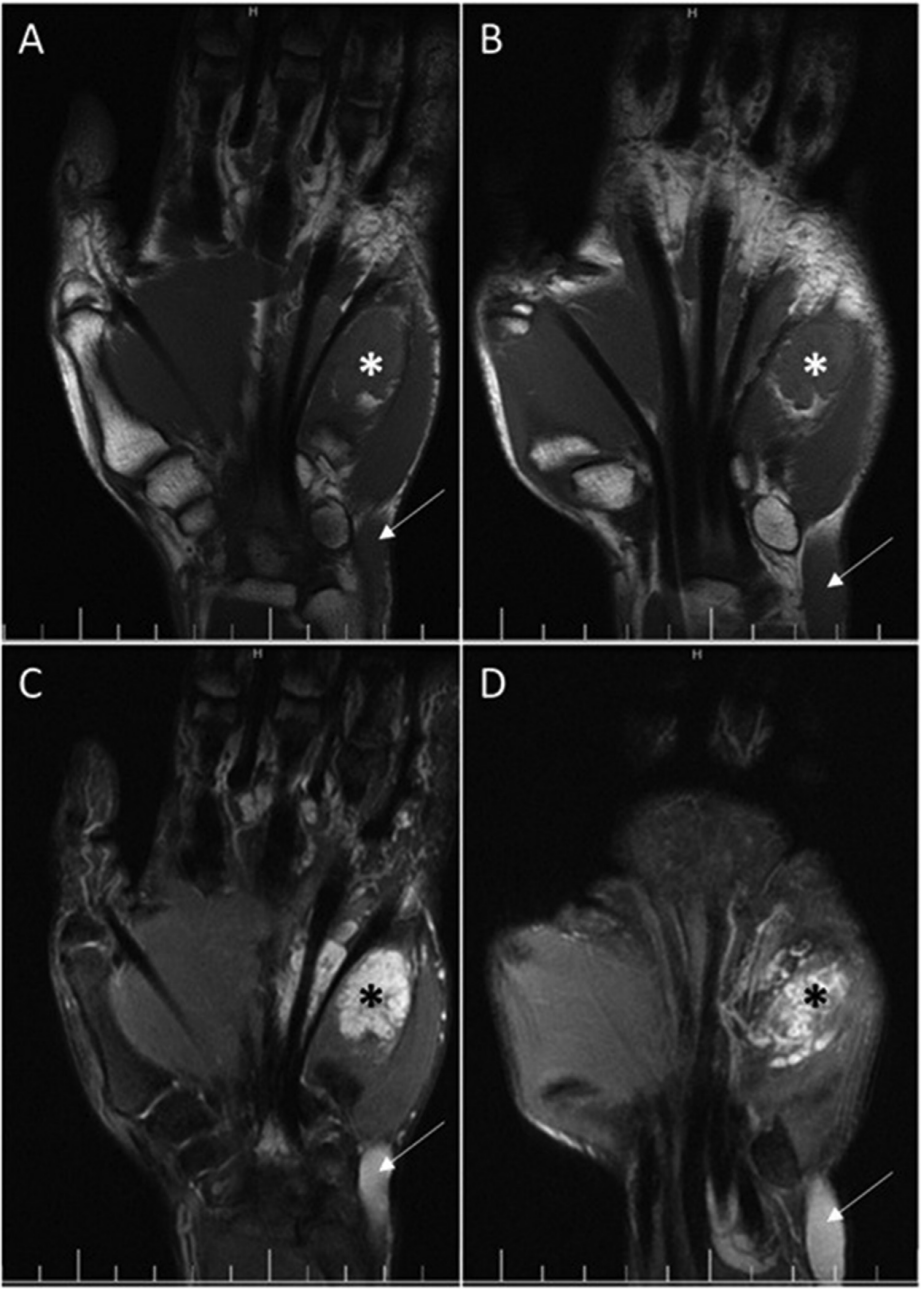
Coronal T1 weighted (A, B) and fat-suppressed T2-weighted (C, D) MR images of the hand and wrist. Soft tissue mass with low signal intensity on T1 weighted images and high signal intensity in T2 weighted images is seen around the flexor tendons (white asterisk). Internal hyposignal areas indicating phleboliths are seen in the palmar region (black asterisk in C and D). Aneurysmal dilatation of the basilic vein is noted (arrow).

Magnetic resonance imaging (MRI) study revealed a well-circumscribed mass with low signal intensity on T1-weighted images and high signal intensity on fluid-sensitive sequences in the hypothenar region with peri- and inter-tendinous growth around the fourth and fifth flexor digitorum superficialis and flexor digitorum profundus tendons. Small internal hyposignal foci within the mass were noted, indicating calcifications. The imaging findings were in favor of palmar hemangioma with phleboliths. The basilic vein was aneurysmally dilated; draining multiple vessels which were derived from the hemangioma (Fig. 2, Fig. 3, Fig. 4).

Patient underwent venous aneurysm resection and reestablishment of basilic vein continuity, along with resection of palmar mass. Histopathologic examination confirmed the nature of the palmar mass as capillary hemangioma. In 6 months follow-up, the patient did not develop any complications, such as recurrence or movement restriction.

## Discussion

Superficial VA is a rare and unsurveyed entity. Very limited number of cases with basilic vein aneurysms has been reported in the literature [1], [2], [3], [4], [5]. Furthermore, there is only one reported case of concomitant occurrence of basilic vein aneurysm and deep upper extremity hemangioma [6]. Hence, our case is the second report of superficial venous aneurysms in association with deep hemangioma.

Among the wide range of differential diagnosis for a soft tissue mass in the upper extremity, masses of vascular origin are usually overlooked by clinicians. This may lead to inappropriate biopsies and subsequent complications. Our clinical observation emphasizes the need for through imaging evaluation before performing any aggressive procedure over a palpable upper extremity soft tissue mass. Doppler ultrasonography is an inexpensive and accessible method used for initial evaluation of any mass with suspected vascular etiology. In cases of diagnostic challenge, computed tomography (CT scans) and MRI can also be used. MRI and MR venography have been introduced as the preferred imaging modalities for evaluation of vascular tumors and lesions of the hand [6] and can reliably propose the correct diagnosis. As for our case, Doppler ultrasonography readily demonstrated the venous nature of the palpable distal forearm mass, and the possible diagnosis of hemangioma for palmar lesion led to performing MRI to better characterize the size and extension of the lesion.

## Conclusion

This clinical observation highlights the fact that vascular lesions should be considered in the differential diagnosis of any asymptomatic extremity lesion, and thorough imaging evaluation is needed before any aggressive procedure. Also, the co-occurrence of venous aneurysm and vascular tumors (especially hemangiomas) should be kept in mind; so it seems reasonable to lower the threshold for utilization of Doppler ultrasound and MRI in evaluation of lesions with suspected vascular origin.
